# Computational Goals, Values and Decision-Making

**DOI:** 10.1007/s11948-020-00244-y

**Published:** 2020-08-04

**Authors:** Louise A. Dennis

**Affiliations:** Center for Autonomous Systems Technology, Department of Computer Science, University of Liverpool, Liverpool, UK

## Abstract

Considering the popular framing of an artificial intelligence as a rational agent that always seeks to maximise its expected utility, referred to as its goal, one of the features attributed to such rational agents is that they will never select an action which will change their goal. Therefore, if such an agent is to be friendly towards humanity, one argument goes, we must understand how to specify this friendliness in terms of a utility function. Wolfhart Totschnig (Fully Autonomous AI, Science and Engineering Ethics, 2020), argues in contrast that a fully autonomous agent will have the ability to change its utility function and will do so guided by its values. This commentary examines computational accounts of goals, values and decision-making. It rejects the idea that a rational agent will never select an action that changes its goal but also argues that an artificial intelligence is unlikely to be purely rational in terms of always acting to maximise a utility function. It nevertheless also challenges the idea that an agent which does not change its goal cannot be considered fully autonomous. It does agree that values are an important component of decision-making and explores a number of reasons why.

## Fully Autonomous AI

In *Fully Autonomous AI*, Wolfhart Totschnig (2020) argues that the use of the word autonomy in much of the debate around Artificial Intelligence is philosophically weak: it presupposes that any such system possesses a fixed final goal that cannot be changed. He considers a *fully autonomous* agent to be one that can change its goals and then examines the mechanisms by which such goal changes might be effected. He argues that an intelligent system’s ability to change its goals will be based on a complex and nuanced understanding of what those goals are and that this, in turn, will be controlled by the system’s values.

He uses this analysis to critique the argument that weak autonomy provides an assurance that if we can but define the final goal appropriately we need not fear the possible development of a super-intelligent AI that is antithetical to humanity (Bostrom 2014; Yudkowsky 2001). In this commentary I respond to the article from a computer science perspective and will focus on how we can understand the notion of value computationally. I will discuss the relationship between some fixed measurable quantity (that is often understood as a goal in AI systems) and a more abstract notion of goals, and the conceptual gap that arises when the task of creating a complex computational system reduces to that of defining a utility function. I will survey both alternative computational descriptions of goals, and alternative proposals for computational decision-making in AI systems and discuss how they relate to the concepts of rational decision-making and utility maximisation. I will argue that an AI that cannot change its utility function may nevertheless be fully autonomous, but I will also agree that we are highly likely to create AIs that can and will change their utility functions. I will then look at computational models of values and ethics and discuss how they may relate to the concept of values that Totschnig proposes in *Fully Autonomous AI*, agreeing that values are an important component of decision-making. While it is unclear whether we can guarantee that some future AI may be friendly towards us through the appropriate specification of our values (however represented), I argue nevertheless that a better understanding of the interaction of computational values and goals and the incorporation of values into AI systems is important for current uses of the technology.

## Computational Goals

Totschnig considers the concept of a computational goal drawn from a range of successful AI techniques where an AI is either given, or learns, a model of the world in which the outcome of action is scored according to a utility function. The system reasons with the view of maximising its expected utility according to this model. This style of reasoning has its roots in game theory and economics, and ultimately the work of Bentham and Mill. The core idea is that a *rational agent* always seeks to maximise its expected utility. A key to the implementation of this style of goal-directed reasoning is, therefore, the ability to score outcomes and create a predictive model of the utility to be gained from performing some action (or sequence of actions) in any given situation. Totschnig’s initial discussion suggests that the terms *goal* and *utility function* are to be used interchangeably.

The argument for the finality of such a utility function derives from the observation that *even if* a mechanism for altering the utility function exists, if an entity is purely fixated upon maximising this function *and* intelligent enough to predict that some action it takes will alter the utility function, then it will never choose that action since ultimately this would mean the agent would no longer maximise that utility.

Totschnig discusses two examples of this kind of goal from the literature: Bostrom’s famous *maximise paper clips* (Bostrom 2014) and *serve humanity* (a variation on Yudkowsky’s *be friendly towards humanity* (Yudkowsky 2001)). Totschnig, following Petersen (2017), argues that neither of these goals provides a completely objective metric for use in a utility function. He highlights in particular questions how a superintelligent entity might understand the term “paper clip”. Bostrom clearly envisages his maximising paper clips superintelligence as adhering to a rigid and well-defined notion of a paper clip (presumably something manufactured to have certain measurable qualities) while Petersen (and Totschnig) argue that it is difficult to imagine an entity which is both superintelligent and unable to redefine its concept of paper clip into something more general that accounts, for instance, for the purpose of a paper clip. I am inclined to agree that it is difficult to imagine something both superintelligent and so rigid, but I’m not convinced that this failure of imagination is necessarily a non-existence argument.

However, this imaginative gap does reveal a conceptual gap between the technical definition of a utility function (maximisation of some measurable quantity) and the concept of a goal as used in non-technical discourse where it is a more abstract quality—even if that is only to maximise paper clips. The goal of serving humanity makes the problem of this gap acute—for the goal and the utility function to be one and the same, it is necessary to specify the good of humanity as a measurable quantity. Indeed the question of how the good of humanity might be specified in this way is the key to the research project of creating human-friendly AI as defined by Yudkowsky.

### Other Computational Notions of Goal

It should be noted that some branches of AI, particularly those derived from the symbolic reasoning tradition such as AI Planning (Ghallab et al. 2016) and Cognitive Agents (Wooldridge (2002)), have different notions of a goal. Most frequently a goal is a symbolic representation of some state of the world to be brought about. A crucial difference here is that a goal either is, or is not, achieved instead of being some quantity to be maximised. In this tradition, therefore, the goal might be equivalent to *enough paper clips* rather than *maximise paper clips*.

In this tradition, there is not necessarily a “final” goal, and there is literature on how goals may be selected for achievement. However this branch of AI does not meet Totschnig’s criteria for full autonomy either: there is no widely agreed upon mechanism for adding new goals for selection into such a system, any more than there is for an agent that maximises utility to derive a new final utility function.

However, the observation that the field of AI has not necessarily agreed upon how the concept of a goal should be represented computationally, leads us also to ask whether the computational path to intelligence necessarily lies in the use of utility functions.

## Reasoning as the Maximisation of Utility

As well as alternative concepts of goals, there are alternative proposals for how an artificial intelligence might reason. Recent advances, it is true, have been driven by improved algorithms for learning governed by reward mechanisms. These ultimately learn a model which can be used to predict the expected utility of an action. However, even if we have such a learned model, the intelligence may use it in more complex ways than simply calculating and then selecting the action with highest expected utility according to the model. For instance, in resource-bounded settings such as limitations of time or memory (Halpern et al. 2013) selecting an action of high-enough utility calculated quickly may be preferable to spending a long time to calculate the action of highest utility. When concepts of risk and uncertainty need to be taken into account (Buchak 2013), a low-risk low-reward action may be preferable to a high-risk high-reward action even if the expected utility of the high-risk action is higher than that of the low-risk action. Indeed, it is unlikely that any computational system, no matter how superintelligent, will be able to evaluate every possible action in reasonable time and predict the outcome with complete certainty. Similarly reasoning mechanisms based upon symbolic representations and logic—or combinations of these with reward/utility based mechanisms—may choose to treat any action that satisfies some logical expression as preferable to any action which does not (no matter how high the reward of the alternative action).

In these scenarios the argument that an agent will never select an action that changes its utility function, because that would yield a lower expected utility according to the model, begins to collapse. It can be argued that all these mechanisms are, in fact, approximations for a utility function that is multi-objective and/or highly non-linear. An important observation here is that the difficulty of calculating the expected utility and/or creating a good enough predictive model in the face of this kind of non-linearity forces the system to reason using variations on pure utility maximisation. Moreover, it should be observed that *even if* some agent is a pure utility maximising reasoner, unless its model is perfect (which cannot be the case—any perfect model of reality must be as complex as reality itself) then there is always the possibility that the action it selects is not the one that maximises its utility (even though that action did maximise utility in the model). Therefore, although such an agent might never choose an action that it *believed* would change its utility function, it might nevertheless choose an action that *in fact did* change its utility function. The argument that an AI cannot be fully autonomous because it cannot change its utility function rests on an assumption that the AI is not only intelligent but also a perfect reasoner. The combination of imperfect predictive models, highly non-linear utility functions and the difficulty of accurately specifying complex abstract goals could easily lead to the creation of artificially intelligent entities that not only have the means to alter their utility function, but also, on occasion, deliberately choose to use those means. Such AIs might not simply select the action of highest expected utility according to their predictive model.

## What is a Value?

So if an AI may compensate for the inaccuracy and complexity of its predictive model by reasoning using some mechanism that can vary its utility function—what mechanism might it use? Totschnig proposes the use of values which he describes as higher order normative entities where goals are normative entities that derive their prescriptive force from the values.^1^

At first glance, this might encourage us to imagine a hierarchy of utility functions in which the utility functions lower in the hierarchy are continually adjusted according to how well they are maximising the utilities higher up the tree.Hedges et al. (2017) discusses a flexible framework for decision-making by rational agents in which an agent is represented as a *functional*, which selects among decision-making functions and shows how a number of decision-making theories, including utility maximisation, can be modelled by these means. While this theory does not consider adaptation of the decision-making functions chosen between, it is not difficult to imagine some similar structure being used to achieve this effect. So a lower level goal might be our old friend, maximising paper clips, while further up the tree we have serving humanity taking on the role of a value. If, at some point, the agent judges that there are currently enough paper clips in the world, this higher level *functional* selects a new utility function to maximise the quantity of some different object judged to be of benefit. Totschnig would argue, I believe, that such a hierarchy still lacks full autonomy—whatever utility function is being used at the root of this tree is the final goal which may not be altered or dropped. It is not entirely clear, in the absence of philosophy settling the question of the meaning of autonomy,^2^ whether a rational agent which seeks to maximise the good of humanity by flexibly adopting and dropping a variety of short-term goals such as maximising paper clips, could genuinely still be described as possessing only weak autonomy. Certainly its behaviour is likely to be such that many lay people would describe it as fully autonomous. However, for the moment, let us accept that a higher-order utility function or *functional* does not capture the concept of a value.

The alternative to a functional or numeric representation of values is a symbolic representation. The field of machine ethics has considered a variety of mechanisms by which some form of symbolic reasoning representing the ethics of a situation might guide or constrain goal-directed reasoning. Arkin et al. (2012), as an example, consider an *ethical governor* that can veto and guide the targeting decisions of an autonomous weapon system according to symbolic representations of the laws of war and the rules of engagement. While this system does not in any way enable the system to change its goal, it is an example of how a symbolic expression of ethics (or values) can be integrated with more numeric styles of decision-making. Bremner et al. (2019) extend this idea to one in which the ethical reasoning cannot only veto suggestions from the goal-directed reasoning but direct that reasoning to consider new options—not quite forcing it to select new goals but rather to evaluate more actions. It is not difficult to imagine a similar mechanism requiring the underlying system to choose a different utility function. Might values therefore be symbolic entities that enable the adaptation of goals depending upon how well those goals are currently perceived as conforming to those values?

Rossi and Mattei (2019) advocate the construction of *ethically bounded AI* in which goal-directed decision-making is constrained by values-based reasoning. They consider not only symbolic approaches such as Arkin’s but also data-driven approaches in which an agent learns two reward functions one of which models a goal and the other ethical constraints (Balakrishnan et al. 2019) and decision-making then mediates between these two utility functions.^3^

The field of AI, therefore, already has a number of computational accounts of values. Can any of these play the role of values in Totschnig’s argument? Totschnig discusses a number of routes by which values could arise. These all assume that a superintelligent AI will, by virtue of its (super)intelligence learn or evolve these values (or alternatively deduce that values are arbitrary). In some cases these values are immutable once acquired—they are normative facts or constitutive of intelligence and in others they are mutable. Therefore, a fully autonomous AI can have its options limited by values (if those values are immutable). It isn’t entirely clear whether Totschnig believes that values must necessarily be self-acquired or the fact that this is a feature of each of his proposed mechanisms is incidental. However, if we assume that values are derived or discovered by an intelligence for itself then we do, indeed, lose the ability to shape those values with certainty and thus any mechanism to guarantee that the resulting intelligence is human-friendly. If, on the other hand, values can be imposed by ourselves in order to ethically bind the AI, then we can potentially have a fully autonomous friendly AI—always assuming we can accurately specify our values. It should be noted that even attempts to derive single utility function expressions of serving humanity reference the concept of values (see for instance the discussion in Yudkowsky (2011)) and anticipate such a function will be highly complex and non-linear. Again, I would argue that such system would have considerable flexibility in reasoning and behaviour and will appear far more autonomous than Bostrom’s paper clip maximisation AI. Nor does it seem logical that the distinction between fully autonomous and weakly autonomous AI should rest upon whether its values are represented symbolically or not.

While the precise computational formulation of values and their interaction with goals is unclear, and thus so is the extent to which they can enable full autonomy and provide us with guarantees of the friendliness of any AI, it is nevertheless widely accepted that as AIs become more powerful, their decision-making must account both for values and goals.

## Conclusion

*Fully Autonomous AI* makes three main claims:

Firstly, it claims that AI systems that are constructed to maximise some fixed well-defined value, modelled as a goal, lack full autonomy. While such a system is free to apply ingenuity to the selection of the means by which it achieves something, it has no autonomy over selecting the ends. In order for an agent to be considered fully autonomous it must be able to change its goals.

Secondly, it argues that values allow agents to interpret and choose goals. It doesn’t specify in a computational sense what these values are but leans towards definitions that imply that such values arise out of the existence of intelligence and away from a suggestion that values can be used as a mechanism to program or shape the development of intelligence.

Lastly, it argues that attempts to mitigate the dangers of superintelligence by choosing appropriate goals are doomed to failure because a genuine intelligence will be able to change, adopt and drop its goals based upon values over which humanity may have no control.

The question of what constitutes full autonomy is philosophical but it is incontrovertible that a definition of autonomy which is constrained to a goal as straightforward as maximising paper clips seems very weak. But, as I have sought to show, our inability to specify more abstract goals in terms of simple measurable quantities does not mean that variations on the kinds of techniques currently in use for reasoning with these simple goals cannot be applied to more abstract notions. It is not obvious that reasoning that involves more complex utility functions (and which will therefore flexibly adopt and drop a wide range of behaviours and sub-goals according to context) cannot be fully autonomous even if that utility function does not change.

However, the argument that an intelligent rational agent will never take an action that will alter its utility function is dependent upon such an agent having a perfect predictive model and no such agent can exist—in part to mitigate this problem a number of sophisticated proposals exist for reasoning with utility functions that do not simply seek to select the action that maximises expected utility according to the model. Therefore, even if an agent with a fixed final goal cannot be considered fully autonomous, we may well create artificial intelligences that are fully autonomous because they do not act with pure rationality according to their predictive model. In these systems, Totschnig’s concerns about whether the beneficence of the intelligence can be guaranteed through careful construction of the goal remains.

Among such approaches to artificial intelligence there is considerable interest in the modelling of values in computational systems and their interactions with goals. It is not obvious whether these proposals would count as values in the manner Totschnig seeks to use them. In general, computational values are used to constrain goal-based reasoning and not as a driver for the adoption of goals, but there is no reason in principle why they could not be used in this way and the concept opens up a number of interesting research questions. These include how such a mechanism might work, and whether such a mechanism would ultimately incorporate values into a single utility function or *functional* and whether such values can be made immutable (and so allow us to guarantee beneficence of an AI through the appropriate specification of values). If these values are not immutable then we must continue to concern ourselves with whether an AI’s values always align with our own.

Perhaps more importantly, and certainly more immediately, our current lack of understanding about how to adequately program behaviour that can flexibly adopt and drop goals is one of the key limitations to our ability to take artificial intelligence to the next level. Values might provide a route to this greater flexibility. Technologically speaking, it may turn out that human values will ultimately be expressible in terms of utility functions or similar computational mechanisms. At the moment however, mechanisms for doing this are not well understood, and there is a conceptual gap between our ideas of value and the concepts we typically use for goals in artificial intelligence. Quite aside from any concerns about superintelligence, it is important that we develop a better understanding of the relationship between values and computational goals. We are now seeking to deploy AI in a wide variety of circumstances where their behaviour interacts with human value systems—obvious examples are domestic and healthcare robots (where values such as respect for human autonomy, dignity, safety and privacy all interact) and the AI based systems we employ to mediate our online experiences (where values such as privacy, community, fairness and truth all interact). A good computational account of values and the ways in which they interact with computational goals is therefore urgently needed.

## Footnotes

1 Hence the tight coupling between goals and utility functions is presumably relaxed.

2 Or, alternatively, choice or free will.

3 Arguably, once again, this is an attempt to approximate a non-linear overall utility function.

## References

[CR1] Arkin RC, Ulam P, Wagner AR (2012). Moral decision making in autonomous systems: Enforcement, moral emotions, dignity, trust, and deception. Proceedings of the IEEE.

[CR2] Balakrishnan, A., Bouneffouf, D., Mattei, N., & Rossi, F. (2019). Incorporating behavioral constraints in online AI systems. In* Proceedings of the 33rd AAAI conference on artificial intelligence (AAAI)*.

[CR3] Bostrom N (2014). Superintelligence: Paths, dangers, strategies.

[CR4] Bremner P, Dennis LA, Fisher M, Winfield AF (2019). On proactive, transparent and verifiable ethical reasoning for robots. Proceedings of the IEEE special issue on Machine Ethics: The Design and Governance of Ethical AI and Autonomous Systems.

[CR5] Buchak L (2013). Risk and rationality.

[CR6] Ghallab M, Nau D, Traverso P (2016). Automated planning and acting.

[CR7] Halpern JY, Pass R, Seeman L (2013). Decision theory with resource bounded agents. Topics in Cognitive Science.

[CR8] Hedges J, Oliva P, Sprits E, Winschel V, Zahn P, Rothe J (2017). Higher-order decision theory. Algorithmic decision theory.

[CR9] Petersen S, Lin P, Jenkins R, Abney K (2017). Superintelligence as superethical. Robot ethics 20.: From autonomous cars to artificial intelligence.

[CR10] Rossi, F., & Mattei, N. (2019). Building ethically bounded AI. In* Proceedings of the 33rd AAAI conference on artificial intelligence (AAAI)*.

[CR11] Totschnig W (2020). Fully Autonomous AI. Science and Engineering Ethics.

[CR12] Wooldridge M (2002). An introduction to multiagent systems.

[CR13] Yudkowsky E (2001). Creating friendly AI 1.0: The analysis and design of benevolent goal architectures.

[CR14] Yudkowsky E, Schmidhuber J, Thórisson KR, Looks M (2011). Complex value systems are required to realize valuable futures. Artificial general intelligence: 4th international conference, AGI 2011.

